# Cancer in Hispanic communities: Lower rates, larger gaps, and the work ahead

**DOI:** 10.3322/caac.70115

**Published:** 2026-10-20

**Authors:** Narjust Florez, Bárbara Segarra‐Vázquez

**Affiliations:** ^1^ Dana‐Farber Cancer Institute Harvard Medical School Boston Massachusetts USA; ^2^ School of Health Professions Medical Sciences Campus University of Puerto Rico San Juan Puerto Rico USA

## Abstract

Lower overall cancer rates in Hispanic people hide excess hepatocellular, gastric, and cervical cancer, a rising burden in women, and widening gaps in coverage, language, and trust. Research conducted with Hispanic communities, rather than simply about them, is essential.

Cancer is once again the leading cause of death among Hispanic people in the United States.^1^ In this issue of *CA: A Cancer Journal for Clinicians*, Saka and colleagues estimate that 229,900 Hispanic people in the continental United States and Hawaii will be diagnosed with cancer this year, and 54,200 will die from it.^1^ Their report is the most complete look yet at cancer in our community, and it should change how we practice and conduct research in this often‐forgotten population. Hispanics have lower cancer incidence than non‐Hispanic Whites and Asians. That is the finding most readers will remember, and, as the authors themselves warn, it marks large disparities in cancers for which risk reduction is possible. The Hispanic population advantage rests largely on lower tobacco use rates and traditional diets largely based on vegetables and legumes; however, Hispanics face double rates of liver and gastric cancer, cholangiocarcinoma, 36% higher cervical cancer rates, and greater than 60% higher incidence of acute lymphocytic leukemia.^1^ Much of this excess is preventable, linked to hepatitis C and *Helicobacter pylori* infection, obesity, and poor access to cancer screening. Only 56% of Hispanic adults aged 45 years and older are up to date with colorectal screening, versus 67% of White adults.^1^

## ONE WORD, MANY COMMUNITIES

Saka et al. acknowledge that registry data cannot be separated by country of origin and is the report’s most important limitation.^1^ The term Hispanic includes people from more than 20 countries with different migration stories, and where each community settles in the United States affects its cancer risk and access to care.^2^ The aggregate tells one story, but the details tell many. Dominicans and Puerto Ricans are concentrated in New York, New Jersey, Florida, and Massachusetts; Salvadorans and Guatemalans in California and the Washington, District of Columbia, area; and Cubans and Venezuelans in South Florida.^3^, ^4^, ^5^ Furthermore, growth has spread to new destinations in the South and Midwest, where health systems have little to no experience treating Hispanic patients.^2^, ^5^ An Hispanic patient in Boston and one in Los Angeles may have very little in common, so outreach, interventions, and research must fit each community. Cancer incidence among people of Puerto Rican origin was nearly twice that of people of Mexican or Central American origin in one cohort,^1^ and mortality is 15% higher in US‐born Hispanic men than in those born abroad.^6^ Survival is also likely overestimated because of loss to follow‐up due to many Hispanics returning home for end‐of‐life care, which helps create the so‐called Salmon bias, causing their deaths to be unrecorded in the United States.^1^, ^7^

## A GROWING BURDEN IN HISPANIC WOMEN

Cancer incidence in Hispanic women has risen until it essentially equals that in Hispanic men, with the male‐to‐female ratio falling from 1.40 in 2002 to 1.01 in 2022; and, among adults younger than 50 years, Hispanic women have more than twice the cancer rate of men.^1^ Currently, breast cancer incidence is growing the fastest among young Hispanic women (aged 20–49 years), at 2.4% per year. When a woman who is raising children and caring for aging parents is diagnosed with cancer, the whole family is affected, with consequences that reach child survival, schooling, and the family’s socioeconomic advancement.

In lung cancer, the incidence in Hispanic women is declining at one half the pace of White women.^1^ In Florida, 35% of lung cancers in Hispanic women occurred in never tobacco users versus 12% in White women,^8^ suggesting risk factors beyond tobacco use. Lung cancer screening rests only on tobacco use history,^9^, ^10^, ^11^ excluding other risk factors, and contributes to nearly one half of Hispanic patients with lung cancer being diagnosed with metastatic disease.^10^, ^11^ We need screening strategies for people who never smoked, and Hispanic patients must help build them. The EQUAL study, for example, is testing a blood‐based approach for the early detection of EGFR‐positive lung cancer in Hispanic and Asian populations with little or no tobacco exposure.^12^

In this report, the incidence data ended in 2022, and the mortality data ended in 2024,^1^ thus not capturing the conditions Hispanic families face today. Hispanic people have the second highest uninsured rate in the country after Native Americans, and one in four Hispanic adults aged 18–64 years has no coverage, three times the rate in White adults.^1^ Spanish is the second most spoken language in the United States, yet nearly one in four Hispanic people who speak it at home speak English not well or not at all, and many never meet a clinician who speaks their language.^1^ The authors link later stage diagnosis to these gaps, perceived discrimination, and fear related to documentation status.^1^

In January 2025, federal policy that had kept immigration enforcement out of hospitals and clinics was rescinded^1^; and, by late 2025, the share of immigrant adults who skipped or postponed health care had risen from 22% to 29%, with nearly one half of likely undocumented adults avoiding medical care.^13^, ^14^ The 2025 reconciliation law is projected to add 10 million uninsured people by 2034, mostly through Medicaid changes.^15^ In oncology, delay can mean the difference between curative‐intent and palliative treatment. In Puerto Rico, where cervical, prostate, and early onset colorectal cancer rates (3.8% per year) are higher than in other US Hispanic populations and 37% of residents live in poverty, these pressures fall on a health system strained by hurricanes.^1^, ^16^

## WHAT IS NEEDED NOW?

Clinicians need to understand the heterogeneity that exists within the Hispanic community and look past traditional risk factors to the patient in front of them, whose country of origin, nativity, and years in the United States shape their cancer risks. Understanding Hispanic heterogeneity is not only a matter of equity but of better science. Furthermore, cancer centers need clear policies on immigration enforcement in clinical areas and need to tell patients what information is shared and with whom. Policymakers should preserve coverage for low‐income families and screening programs, such as the National Breast and Cervical Cancer Early Detection Program, and should conduct research in the populations that bear the highest burden.^17^ Describing disparities is no longer enough. We need implementation research that takes what already works—promotoras de salud, faith‐based communities, and Spanish‐language media—into the places where Hispanic families live and work and evaluates whether screening and enrollment improves.^18^, ^19^ However, that research must be built on long‐term relationships by asking communities how they want to be reached and what they want to learn. A program that leaves when the grant ends erodes the trust that many investigators like us have spent years building in the Hispanic community.

The time is now to study cancer in Hispanics with the same rigor we bring to any other population.^20^ Two of every three Hispanic people were born in the United States; births rather than immigration now drive population growth, and US‐born Hispanics have higher cancer incidence than those born abroad, so this burden will continue to grow.^1^, ^2^ That means cohorts large enough to separate origin groups and research on lung cancer in women who never smoked, on early onset cancers, and trials that enroll Hispanic patients in proportion to the one in five Americans who share our heritage.^1^ We also need genomic studies that include Hispanic patients and registry data linked to origin, nativity, and language. Every Hispanic population belongs in this work: Afro‐Latinos and Indigenous patients, some of whom speak neither Spanish nor English; residents of Puerto Rico, whose cancer data lag years behind; and recent immigrants, who rarely appear in our cohorts.^1^ The political environment changes with each administration and may make this work harder for a time; however, Hispanic people are here to stay. Hispanic workers are the largest share of the labor force in the country, and their health is the health of the United States. Detrás de cada número hay una persona. Behind every number is a person, and a family waiting for us to act.

## CONFLICT OF INTEREST STATEMENT

Narjust Florez reports institutional research funding from AstraZeneca, Boehringer Ingelheim, Daiichi‐Sankyo, and Genentech; grants/contracts from Daiichi‐Sankyo Foundation of Life Science, Genentech Foundation, and Janssen Biotech, Inc.; personal/consulting or advisory fees from Amgen, AstraZeneca, Boehringer Ingelheim, Catalyst Pharmaceuticals, Inc., Daiichi Sankyo Company, Daiichi Sankyo Foundation of Life Science, Inivata, Inc., Janssen Biotech, Inc., Janssen Pharmaceuticals, Jazz Pharmaceuticals, Johnson & Johnson, Merck, Mirati, Neogenomics, Novartis, Novocure Inc., Nuvalent, Pfizer, Regeneron, Regeneron Pharmaceuticals, and Takeda Oncology; and support for other professional activities from Clinical Care Options, CME Outfitters, Genentech Foundation, IDEOlogy Health, OncLive/MJH Life Sciences, Nuvation Bio, PER, and Precisca outside the submitted work. Bárbara Segarra‐Vázquez disclosed no conflicts of interest.
